# Post-Takeover Proficiency in Conditionally Automated Driving: Understanding Stabilization Time with Driving and Physiological Signals

**DOI:** 10.3390/s24103193

**Published:** 2024-05-17

**Authors:** Timotej Gruden, Sašo Tomažič, Grega Jakus

**Affiliations:** Faculty of Electrical Engineering, University of Ljubljana, Tržaška cesta 25, 1000 Ljubljana, Slovenia; saso.tomazic@fe.uni-lj.si (S.T.); grega.jakus@fe.uni-lj.si (G.J.)

**Keywords:** takeover, stabilization, conditionally automated driving, driving simulator, user study, physiology

## Abstract

In the realm of conditionally automated driving, understanding the crucial transition phase after a takeover is paramount. This study delves into the concept of post-takeover stabilization by analyzing data recorded in two driving simulator experiments. By analyzing both driving and physiological signals, we investigate the time required for the driver to regain full control and adapt to the dynamic driving task following automation. Our findings show that the stabilization time varies between measured parameters. While the drivers achieved driving-related stabilization (winding, speed) in eight to ten seconds, physiological parameters (heart rate, phasic skin conductance) exhibited a prolonged response. By elucidating the temporal and cognitive dynamics underlying the stabilization process, our results pave the way for the development of more effective and user-friendly automated driving systems, ultimately enhancing safety and driving experience on the roads.

## 1. Introduction

In recent years, the advent of conditionally automated vehicles (Society of Automotive Engineers—SAE Level 3 [1]) has promised transformative shifts in transportation, offering the potential to enhance road safety, efficiency, and convenience [2,3,4,5]. Conditionally automated driving systems, which delegate all driving tasks to the vehicle only when the appropriate conditions are met but still require human supervision, promise significant potential to alleviate driver fatigue, reduce human error, and mitigate traffic congestions. However, realizing these benefits hinges upon a critical factor: the seamless transition of control between the automated driving mode and human operation, commonly referred to as the “takeover” process.

While conditionally automated vehicles offer the allure of a more relaxed driving experience, they also introduce unique challenges, particularly during the handover of control from the automation to the human driver. Maggi et al. define takeover as “the process with which one agent takes back control of part or all of the dynamic driving task” and handover as “the parallel process with which one agent relinquishes part or all of the dynamic driving task” [6]. This transition phase represents a period of increased risk and uncertainty, where drivers must swiftly re-engage in the driving task to ensure safety. Research has highlighted the potential pitfalls associated with takeover situations, including delayed reaction times, reduced situational awareness, and increased likelihood of accidents [7,8,9,10,11,12,13]. Multitasking, for example, is a problem already present in conventional vehicles and is only likely to increase with the introduction of conditionally automated vehicles [14,15].

A crucial but understudied aspect of takeover scenarios is the concept of stabilization time: the time it takes a driver to regain full control and exhibit consistent, safe driving behavior following a transition from automated to manual driving. Understanding stabilization time is paramount for optimizing the design and implementation of conditionally automated systems, as it directly impacts the safety and user experience of such technologies.

In this article, we aim to address the pressing need for a comprehensive investigation of stabilization time in conditionally automated driving environments. With driving simulator user studies and advanced physiological signals analysis, we seek to determine and elucidate the factors influencing stabilization time. By shedding light on the temporal and cognitive dynamics during the takeover process, our research seeks to enhance the efficacy and acceptance of conditionally automated vehicles, paving the way for safer and more seamless integration into our transportation systems.

### 1.1. Related Work

The integration of automated vehicles into existing transportation systems necessitates a comprehensive regulatory framework to ensure their safe and responsible use. The planned amendment to the Vienna Convention on Road Traffic [16] provides that a takeover can be requested at any time either by the vehicle or by the driver. According to the SAE J3016 standard [1], conditionally automated vehicles must provide “the sustained and operational design domain-specific performance of the entire dynamic driving task under normal operation with the expectation that the user is receptive to automated driving-system requests to intervene, as well as to relevant system failures in other vehicle systems, and will respond appropriately.” This does not require the driver to monitor the journey while the system is in operation. Working party no. 29 of the World Forum for Harmonization of Rules on Vehicles and their Systems and Units proposes amendments to the United Nations’ regulations on automated lane keeping systems (UN Regulation No. 157 and UN Regulation No. 79) [17]. In this context, it was noted that the rules for fully automated systems which can decide on the appropriate maneuver to take in relation to the traffic situation have not yet been finalized [18]. The International Organization for Standardization (ISO) offers two standards on this topic: ISO 26262-1:2018 (Road vehicles—Functional safety) [19] and ISO 21448:2022 (Road vehicles—Safety of the intended functionality) [20]. These two standards complement each other and together form the basis for safety and advanced support systems in first- and second-level automated vehicles, but do not interfere with further levels of automation. Therefore, the transition to further levels of automation and the associated problem of the takeover process are still under research.

#### 1.1.1. On Takeover Process

If proper conditions are met, e.g., a dedicated road infrastructure is in place, normal weather and visibility conditions prevail, advanced driver assistance systems (ADAS) are working, etc., a conditionally automated vehicle drives in fully automated mode. Meanwhile, the driver can engage in other tasks, such as writing emails, reading a book, etc. In case of a predictable situation (e.g., exiting a highway) or an unpredictable situation (sensor failure, traffic collision, road works, extreme weather conditions, etc.) that the vehicle cannot safely manage, it alerts the driver and the driver must take over. Normally, a takeover (TO) process starts with a takeover request (TOR) issued by the vehicle.

The timing of the TOR is one of the crucial parameters of every TO process [8]. The takeover lead time (TORlt) is defined as the time interval between the TOR and the anticipated situation requiring a TO, e.g., a collision or the end of the road. Researchers around the globe use different TORlts in their research. Sanghavi et al. showed that drivers’ reactions were fastest when using three seconds as the TORlt, but the safest and least demanding reactions were provoked with a TORlt of seven seconds [21]. Shi et al. similarly showed that drivers elicited the overall best reactions when using a TORlt of six seconds [22], while Tan and Zhang concluded that drivers’ situational awareness was best when using a TORlt between 16 and 30 s [23]. Eriksson and Stanton conducted a literature review and found that the most commonly used TORlts among the studies reviewed were 3, 4, 5, 6, 7, and 9 s [24].

After the vehicle initiates a TOR, the driver is responsible for taking over in a decent time, referred to as reaction time (RT). It is most commonly measured from the TOR until the driver turns the steering wheel more than two degrees (steering reaction time) or presses the brake pedal more than 10% (brake reaction time) [25]. For a deeper understanding, RT could be further divided into shorter intervals, e.g., for gaze switching from a secondary task to driving, gathering information (becoming aware of the situation), and deciding on the appropriate action [26]. According to the situation awareness (SA) theory [27], which is often used in vehicular human–machine interaction planning [28,29], achieving SA entails perception of environmental elements (SA level 1), comprehension of their significance (SA level 2), and the ability to anticipate their future status (SA level 3). Therefore, some researchers divide the driver’s readiness to take over the vehicle into visual, mental, and physical readiness [9,26].

Regarding the user interfaces (UIs) for issuing the TOR, researchers first explored the appropriate modalities. Auditory interfaces (beeps) are known to provoke the fastest reactions [30], while tactile patterns [31,32,33] and ambient light [34,35] reduce drivers’ effort and increase their situational awareness. Most concluded that multimodal user interfaces should be used when issuing a TOR [36,37]. If researchers’ conclusions are consistent in terms of UI modality, we did not find this to be the case for the different types of stimuli. Auditory beeps represent the most often used stimuli [38,39,40]; Stojmenova et al. reported that a pure tone of 4000 Hz provoked the fastest reactions during driving experiments [40]. Politis et al. recommend abstract visual cues in non-urgent situations and additional auditory warning for increased urgency of the situation [38]. Among tactile interfaces, meaningful stimuli (e.g., different patterns) generally do not improve takeover performance, but could increase the driver’s mental load [39,41]. Wu et al. showed that including the recommended steering direction in the TOR increases TO safety by decreasing the reaction time and reducing mental workload [42]. Additionally, Kraut et al. recently reported that assertive TO requests lead to shorter reaction times without finding any other effect on driver performance, stress, and subjective perceptions [43].

While the driver is taking over the demanding driving task, they can already observe the road to become aware of the situation as soon as possible. Various support systems besides mere TOR mechanism would be of additional benefit due to lack of time, possible fatigue, bad weather conditions, and other factors that could occur during the TO [44]. Recent related studies considered extended reality (XR) technologies in the first few moments of TO [44], steering wheel with torque guidance mechanism [45], and additional gradual braking systems [46]. It seems that strategies which monitor the driver’s reactions and adapt the user interface accordingly should be used to achieve the best TO performance [46,47].

#### 1.1.2. On Post-Takeover Stabilization

Overall, a proper takeover that ensures a safe and efficient transition of control between the automated system and the human driver involves a combination of timely response, attentiveness, readiness, smooth transition, correct actions, and awareness of the system’s limitations. However, this process does not end with taking over the vehicle, i.e., by grabbing the steering wheel or applying the brakes, as the driver must resolve the critical situation that led to the initiation of a TO procedure and continue driving the vehicle manually. We refer to this phase of adaptation back to manual driving as achieving post-takeover stabilization or simply stabilization.

To account for stabilization, Shull et al. [48] and Ma et al. [49] recommended multi-stage TO requests. However, Butmee et al. [50] and Pipkorn et al. [51] showed that stabilization is almost impossible to achieve in some cases and therefore recommended automatically stopping the vehicle without even trying to issue a TOR. Gruden et al. noted in their study [39] that some drivers only took over the vehicle (i.e., applied the brake pedal) but were unable to perform any other action to prevent collisions. Therefore, they proposed a TO UI that helps the driver to take over the vehicle by providing additional warnings to minimize the stabilization time [46].

Determining the required stabilization time is also necessary for planning how often a driver can perform a TO; stated otherwise, this is how long a manual or automated driving must last before initiating a transition back. Feldhütter et al. explored how the duration of automated driving affects TO performance and concluded that there was no difference in performance when drivers had 5 or 20 min between consecutive TOs [52]. This could be understood as a hint that the stabilization phase after the transition of control might have already concluded prior to new TORs in both cases. Bourrelly et al. showed that longer periods of automated driving lead to poorer performance [53]. However, this is more probably a consequence of fatigue or some other phenomenon rather than stabilization after TO. On the other hand, Zhang et al. observed a degraded driving performance in terms of lane control for more than five minutes after TO [54]. Kim et al. measured the stabilization time after TO as reported by the drivers, i.e., the driver had to say “stable” after taking over and safely handling all driving functions [55]. They reported mean stabilization times of 11.5 s, 22.7 s, and 28.7 s for three consequent studies, found that stabilization time was longer when the accident occurred on the road in front of the vehicle compared to accidents in the oncoming lane, and observed some differences in age and gender. Gaspar et al. proposed adapting the TORlt based on whether drivers had achieved sufficient stabilization in previous TO attempts [56]. Riahi Samani and Mishra examined how long the TOR effect lasts and concluded that the first ten seconds after the TOR carry the most significant information, while the effects significantly reduce after 20 s [57]. Choi et al. showed that cognitive and visual load due to secondary tasks have different effects on stabilization after the TO [58]. Cognitive load increased the time from TO until resolving the situation (i.e., lane change), while visual load increased the steering wheel angle variability after the situation was already resolved, alluding to different effects of secondary tasks on stabilization time after a TO.

#### 1.1.3. On Driving-Related and Physiological Predictors of Stabilization

As stabilization could only be achieved after the TO itself, not all common metrics for assessing TO performance could be used (e.g., reaction time or minimal time to collision do not contain any information on post-takeover stabilization). On the other hand, metrics that evaluate overall driving style could be compared within short intervals following the TO. The most obvious would therefore be to measure lane deviation/winding and determine when it stops. This could be achieved by observing steering wheel angle variability, lateral accelerations, or lane position deviation [59,60,61,62,63,64,65]. In the previously referred study, Zhang et al. measured the duration of how long the driver was out of their supposed lane per minute and recorded more than 10 s per minute for more than five minutes following the TO in about 25% of drivers [54]. Riahi Samani and Mishra calculated the driving-behavior-related parameters (maximum acceleration/deceleration, standard deviation of lane position, headway, maximum/minimum speed) in time windows of 10 s [57]. Their Multilevel Mixed-Effect Parametric Survival Models analysis showed that the first ten seconds after the takeover request contained the majority of information, while the probability of unsafe behavior significantly reduced only 20 s after the TOR. Choi et al. measured steering angle variability during two consecutive intervals: between TO and lane crossing and in the first five seconds after lane crossing [58]. Their conclusions differed depending on the type of secondary task. Kim et al. recorded stabilization time by instructing the drivers to say “stable” when they were fully capable of driving [55]. The stabilization times they measured in three experiments varied between 10 and 30 s, with longer stabilization times in scenarios involving an accident on the road or multiple vehicles in the vicinity.

Although stabilization in terms of driving-related predictors was almost always measured using lane deviation, it was not often compared to drivers’ physiological stabilization or arousal. The duration of physiological responses to TO, such as pupil size, gaze dispersion, heart rate, and skin conductance stabilization was rarely measured. In general, the most commonly measured physiological signals during driving include (1) eye-tracking data such as pupil size, blink rate, horizontal gaze dispersion [25,65,66,67,68,69,70]; (2) heart rate or heart-rate variability [71,72,73,74]; and (3) phasic skin conductance [60,70,72,74]. It is also worth noting that the range of physiological responses during TO vary significantly between individuals [74]. Feldhütter et al. reported slower gaze responses in TOs after 20 min of automated driving than in TOs after five minutes of automated driving [52]. Kerautret et al. reported a long-lasting increase in heart rate after a TO in an emergency situation [75]. Gruden et al. reported on delays and durations of physiological responses to TORs, revealing that pupil diameter had the fastest response with an average duration of about 10 s, while the phasic skin conductance response lasted about 20 s and heart rate almost 60 s [76].

### 1.2. Our Contribution

Achieving post-takeover stabilization seems to be an often-overlooked aspect of TO scenarios. Understanding stabilization time is vital for optimizing the design and implementation of conditionally automated systems. By exploring how drivers adapt to and recover from takeover events, we can inform the development of more intuitive and user-friendly automation interfaces, as well as tailor training programs to enhance drivers’ preparedness for takeover situations. Previous research studies have inadequately addressed the complexity of stabilization time, primarily due to the following pitfalls:

- Limited scope: Many studies have focused narrowly on the time it takes a driver to physically regain control of the vehicle, such as braking reaction time or steering wheel movement, neglecting the broader cognitive and behavioral aspects that influence stabilization time. Zeeb et al. [77], Kim et al. [78], and Radlmayr et al. [79] have shown that analyzing reaction time alone does not provide sufficient insight into the takeover quality. Moreover, Gold et al. concluded that although some interfaces led to faster reaction times, the drivers’ actions were of poorer quality [7]. This narrow focus fails to capture the full extent of the transition process and its implications for safety.
- Lack of generalization: Many studies were conducted exclusively in controlled laboratory environments, using a predetermined TO user interface. For example, Zhang et al. issued a takeover request with only an audible, spoken warning [54]. Kim et al. did not even report how a TOR was issued in their study [80]. Additionally, almost all of the presented studies issued a TOR as a one-time event, while Gruden et al. [46] showed that TO is a process that should be monitored and that warnings should be adapted to the driver’s reactions. This limits the generalizability of findings and may not accurately reflect the challenges drivers face when driving different vehicles.
- Insufficient consideration of physiological factors: Previous research has often overlooked the role of physiological factors, such as stress, cognitive load, and fatigue, in influencing stabilization time. For example, Riahi Samani and Mishra analyzed driving behavior by measuring only vehicle acceleration, speed, and position [57]. Choi et al. reported numerous driving-related parameters (speed, reaction times, maximal wheel angle, etc.) before and after TO, but only measured drivers’ subjective perception with a single visual analog scale at the end of the driving trials [58]. Similarly, Zhang et al. performed a thorough analysis of driver behavior with driving-related parameters and a questionnaire at the end of the trial, but also included heart-rate variability as the only physiological measurement [54]. Therefore, it is possible that some long-lasting effects on the driver’s state after the TO were overlooked by measuring only vehicle parameters.

In this paper, we aim to address these shortcomings by conducting comprehensive investigations into stabilization time in conditionally automated driving environments. We expand the current scope of knowledge by determining the post-takeover stabilization time with multiple variables using data from studies with different TO user interfaces. Furthermore, our investigation of physiological signals, such as heart rate variability and electrodermal activity, offers novel insights into the cognitive and emotional states underlying post-takeover performance, enriching our understanding of driver behavior in dynamic driving environments. To summarize, the research questions of our study are:

1. How long after the takeover could stabilization be achieved? Could this be before reaching the system limit (e.g., impact), i.e., is it more or less than the provided TOR lead time (6 s on average in the reviewed literature)?
2. Do physiological signals elicit a similar stabilization time as driving-related parameters? Does a driver remain stressed or aroused longer after the TO than could be predicted from vehicle parameters?

The reminder of this article is organized as follows: Section 2 presents the datasets used and our analysis procedure. Section 3 presents the results. The discussion is presented in Section 4, and a brief conclusion can be found in Section 5.

## 2. Materials and Methods

Datasets from two driving simulator user studies conducted as part of the European Union’s Horizon 2020 research and innovation program HADRIAN (Holistic Approach for Driver Role Integration and Automation–Allocation for European Mobility Needs) were combined and used for the analysis. In the first user study, Stojmenova Pečečnik et al. explored four types of head-up displays (HUD) to assist drivers in conditionally automated vehicles [81,82]. In the second user study, Strle et al. evaluated the proposed HUD against a baseline condition in terms of cognitive load (physiological signals analysis) induced by the HUD [83]. Both studies involved similar conditions: a within-subject design, the driving scenarios featured a city road, the drivers were asked to take over the vehicle four times per scenario, and the same driving simulator setup was used. The only difference was the interfaces used for driving assistance and takeover requests. As we seek general results that are valid for any type of takeover user interface, we joined the datasets for analysis.

### 2.1. Technical Set-Up

#### 2.1.1. Driving Simulator

**Figure 1:**
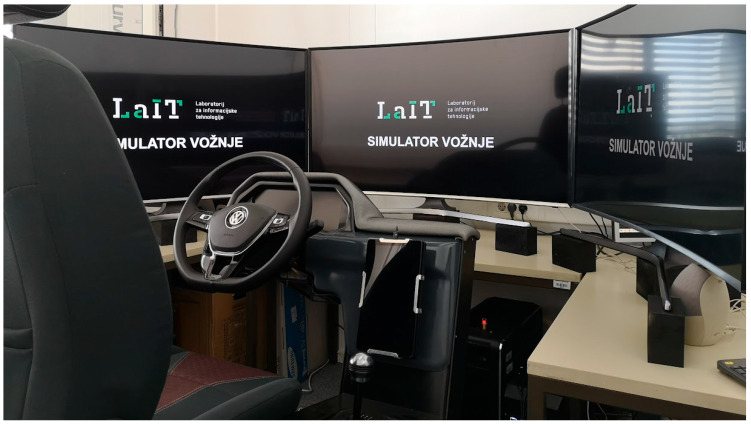
NERVtech^TM^ motion-based driving simulator located at the University of Ljubljana, Faculty of Electrical Engineering.

Both studies were conducted in a NERVtech^TM^ motion-based driving simulator (Nervtech d.o.o., Ljubljana, Slovenia) [84] that consists of three 49″ FullHD curved displays covering the driver’s viewing angle of about 145°, a steering wheel, pedals, a 4-DoF (degrees of freedom) motion platform, and a physical dashboard display; see Figure 1. Using a high-fidelity driving simulator offered several advantages for studying takeover performance (a controlled, safe, risk-free, and standardized environment). Some critical driving scenarios that provide valuable insights into stabilization time could never be safely performed in the real world. In simulators, they can be systematically manipulated and repeated. This level of control also allows for precise measurement of key variables. In addition, driving simulators have been widely used in previous research to study driver behavior, cognitive processes, and performance in various driving tasks. Some studies showed comparable results between studies using real vehicles and motion-based driving simulators, both in terms of physical validity (e.g., vehicle dynamics [85]) and behavioral validity (e.g., car sickness [86]). However, Bellem et al. recommended a motion scaling factor of approximately 50% to 60%, as speed may be underestimated in virtual environments [87]. While we acknowledge that simulators cannot perfectly replicate the complexity of real driving, we believe that our studies have minimized any significant discrepancies between simulated and real vehicles.

The software used to create and play the scenario was AVSimulation’s SCANeR Studio 1.7 [88]. The scenario was developed internally to mimic an urban journey for a person driving from home to work through different parts of the city during the day. It is 13 km long and takes about 16 min. At the beginning, the vehicle was parked on the road with two lanes (one for each direction), and the speed limit was 50 km/h. The surrounding traffic was included to simulate a busy small-town road. After about 3 km there were some crosswalks where pedestrians wanted to cross the road. About 6 km from the starting point, the scenario included a complicated intersection where the road widened to 5 lanes and the driver had to move to the appropriate lane based on the navigation system’s instructions. The journey then continued on a four-lane road (two lanes in each direction). About 10 km from the starting point there was a school area where children crossed the road at crosswalks without traffic lights. After driving 13 km in total, the scenario ended with the driver being asked to park in the street parking lot on the right side of the road, which simulated arrival at the office. During each journey, the vehicle requested four takeover attempts. First, to handle some of the crosswalks; second, to change lanes at the complicated intersection; third, to drive through the school grounds; and fourth, to park the vehicle at the end. Before and after this, the driver was asked to engage the automated driving function. The driver could take over the vehicle by turning the steering wheel or pressing the brake or accelerator pedal.

#### 2.1.2. Head-Up Display and Takeover Requests

**Figure 2:**
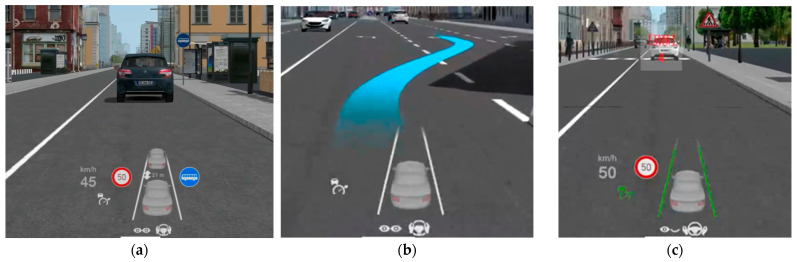
Head-up display (HUD) in the simulated vehicle: (**a**) represents an example of the information displayed on the HUD; (**b**) shows navigation instructions, projected directly onto the road; and (**c**) shows the takeover request notification.

The simulated vehicle featured a head-up display (HUD) designed to assist the driver in monitoring the environment by presenting driving-related information such as current speed, speed limit, safety distance, and the status of assistance systems (see Figure 2a). The amount and location of the presented information varied between trial conditions. For example, some conditions also included navigation information projected directly onto the road (see Figure 2b).

When the vehicle in the automated mode approached its system limits, it issued a TOR five seconds before automated driving would become unavailable. The request was presented with an auditory signal (sine wave, 4000 Hz) and a visual notification with countdown on the HUD (see Figure 2c). At some TORs (when approaching a school area), the TO countdown was already displayed five seconds before the acoustic warning. A period of manual driving followed every TO. When automated driving became available, a synthesized female voice asked the driver to turn on the automated driving function by pressing a dedicated button on the steering wheel lever.

#### 2.1.3. Wearable Sensor Devices

The physiological data were collected using two wearable sensor devices. The eye-tracking data (pupil diameter, gaze) were recorded using Tobii pro glasses 2 [89] with a sampling frequency of 50 Hz. The Empatica E4 wristband [90] was used to record blood volume pulse (BVP) and extract heart rate using the E4’s internal algorithm, galvanic skin response (GSR), and skin temperature. The current heart rate was calculated once per second, while GSR and skin temperature were captured with a sampling frequency of 4 Hz.

### 2.2. Participants and Their Tasks

A total of 30 drivers participated in the first study [82] (16 male, 14 female; aged 23 to 55). As the study had a within-subject design with four trials (types of HUD) and there were four TOs in each trial, this resulted in a total number of 480 TO attempts. 28 drivers participated in the second study [83] (14 male, 14 female; aged 21 to 57). As each participant in the study completed two trials (types of HUD) and there were four TOs in each trial, the study provided 224 TO attempts. In total, we analyzed 704 TOs.

Participation in the study was completely voluntary. Participants were informed that they could stop the experiment at any time without providing a reason. They received a gift voucher of 10 € as compensation for their time. Informed consent was obtained from each participant. The study was conducted in accordance with the code of ethics of the University of Ljubljana which is consistent with the Declaration of Helsinki.

The driver’s primary responsibility was to ensure safe driving continuity. They were asked to drive the vehicle according to the navigation system and get to the destination parking lot. When the automatic driving mode was available, drivers were asked to activate it and engage in a secondary task—a 2048 puzzle game [91] on a smartphone.

Before participating in the study, the detailed procedure was explained to each participant and the conductor collected written informed consent and a demographic questionnaire. Participants began with a test drive to familiarize themselves with the driving simulator and ask questions. Each trial began and ended with manual driving. During the whole trial (four TOs) the experiment was not interrupted. After the trial, participants completed four questionnaires (a custom made Likert scale about the system, a System Usability Scale—SUS [92], a User Experience Questionnaire—UEQ [93], and an Acceptance of Advanced Transport Telematics questionnaire [94]). However, these questionnaires focused on the HUD being tested and not on the takeover procedure [95]. As they served other research purposes, they do not provide information on our research questions (stabilization time) and were therefore omitted from our analysis. More detailed descriptions of the study procedures can be found in Stojmenova Pečečnik et al. [82] and Strle et al. [83].

### 2.3. Variables of Interest

According to related work, driving-related stabilization could be assessed by measurements of lane deviation [54,58]. Among physiological signals, GSR, pupil diameter, and heart rate seem to be good indicators of drivers’ mental or physiological state [74,96,97]. Similar to the way alterations in the GSR signal can indicate the intensity of emotional state, such as happiness or stress [98], pupil diameter directly reflects the driver’s mental load [66], while heart rate and heart rate variability reflect the autonomic nervous system which might indicate a stress response [99,100].

To determine and compare the post-takeover stabilization time we collected the following driving-related and physiological data:

1. Driving-related variables:
  - Winding (standard deviation of steering wheel angle);
  - Speed;
  - Deceleration.
2. Physiological variables:
  - Eyes off-road ratio (E-OFF);
  - Pupil diameter (PD);
  - Heart rate (HR);
  - Phasic skin conductance (SC).

### 2.4. Analysis Procedure

Steering wheel angle, vehicle speed, deceleration, eye gaze, pupil diameter, heart rate, and skin conductance were measured continuously throughout the driving trial. To study post-takeover stabilization, we applied two time windows: one of two seconds and one of five seconds, and we performed a separate analysis for both cases. The time windows (data bins) started at the moment the driver took over the vehicle, i.e., started driving manually, and were calculated every other second (starting at 0 s, 2 s, 4 s, 6 s, etc. after TO). In this way, we obtained non-overlapping windows in the first case, when the window length was two seconds, and overlapping windows in the second case, when the window length was five seconds.

Winding was calculated as the standard deviation of the steering wheel angle for each time window. Additionally, we used the mean speed and deceleration of each time window. Among the physiological variables, we observed eye gaze and determined whether the gaze was directed toward or away from the road. We then calculated the ratio of samples with eye gaze off the road (E-OFF). The mean pupil diameter (PD) and heart rate (HR) for each time window were also calculated. To account for individual differences in PD, we subtracted the value at TO from each subsequent sample of the same driver (shifted the data) so that each PD starts at zero at the TO. Phasic skin conductance (SC) was calculated from the raw GSR signal using the cvxEDA algorithm (convex optimization approach) by Greco et al. [101]. We used the mean phasic skin components in each time window.

The means of the calculated variables over all TO attempts were plotted in the time range from the TO to the time window starting 12 s after the TO (7 windows). We performed repeated measures analysis of variance (RM ANOVA) with Greenhouse-Geisser correction when the sphericity assumption was violated for each variable of interest with the window start time as the factor. If differences between time windows were confirmed, we performed paired-samples *t*-tests for every pair of time windows with Bonferroni adjustment for multiple comparisons. We considered the variable stabilized in a given time window if no differences were observed between that time window and all subsequent windows. An alpha level of 0.05 was used if not stated otherwise.

Data were processed with Python 3.9.12 (anaconda distribution) [102] and analyzed with IBM SPSS Statistics 22 [103].

## 3. Results

The following subsections present the plots of the measured variables for each time window and the statistical analysis of the differences between the adjacent time windows.

### 3.1. Driving-Related Variables

**Figure 3:**
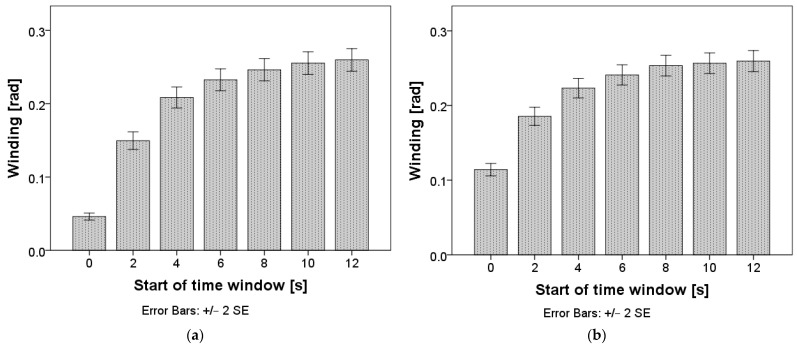
Winding (standard deviation of steering wheel angle) after the takeover. Chart (**a**) represents calculations with a window length of two seconds; chart (**b**) represents calculations with a window length of five seconds.

Repeated measures ANOVA confirmed that differences in winding exist between time windows: F(3.1, 1957.4) = 251.1, *p* < 0.001 for time windows of two seconds, and F(2.1, 1335.6) = 185.8, *p* < 0.001 for time windows of five seconds. The mean values are presented in Figure 3, and the pairwise comparisons can be found in Appendix A, Table A1 and Table A2. Winding stabilized in the time interval starting eight seconds after the TO for both window lengths.

**Figure 4:**
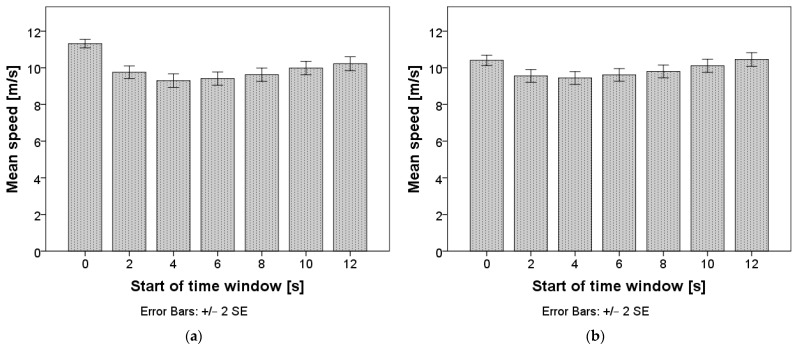
Mean speed after the takeover. Chart (**a**) represents calculations with a window length of two seconds; chart (**b**) represents calculations with a window length of five seconds.

Repeated measures ANOVA confirmed that differences in speed exist between time windows: F(2.1, 1338.0) = 34.9, *p* < 0.001 for time windows of two seconds, and F(1.5, 989.7) = 13.5, *p* < 0.001 for time windows of five seconds. The mean values are presented in Figure 4, and the pairwise comparisons can be found in Appendix A, Table A1 and Table A2. When using a window length of two seconds, speed stabilized only in the time interval starting ten seconds after the TO. The drivers slowed down immediately after the TO and then accelerated until the speed stabilized. When using a window length of five seconds, speed already first stabilized in the time interval starting two seconds after the TO, when the drivers slowed down. Afterwards, the drivers accelerated and the speed stabilized again in the time interval starting eight seconds after the TO.

**Figure 5:**
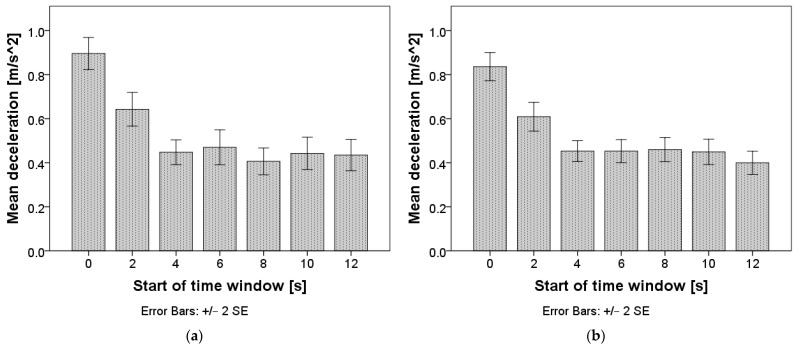
Mean deceleration after the takeover. Chart (**a**) represents calculations with a window length of two seconds; chart (**b**) represents calculations with a window length of five seconds.

Repeated measures ANOVA revealed no differences in deceleration between time windows when using a window length of two seconds F(4.6, 317.9) = 1.01, *p* = 0.410, but revealed statistically significant differences between time windows when using a window length of five seconds F(3.4, 684.4) = 5.46, *p* = 0.001. The mean values are presented in Figure 5, and the pairwise comparisons for the window length of five seconds can be found in Appendix A, Table A2. When using a window length of five seconds, deceleration stabilized in the time interval starting two seconds after the TO.

### 3.2. Physiological Variables

**Figure 6:**
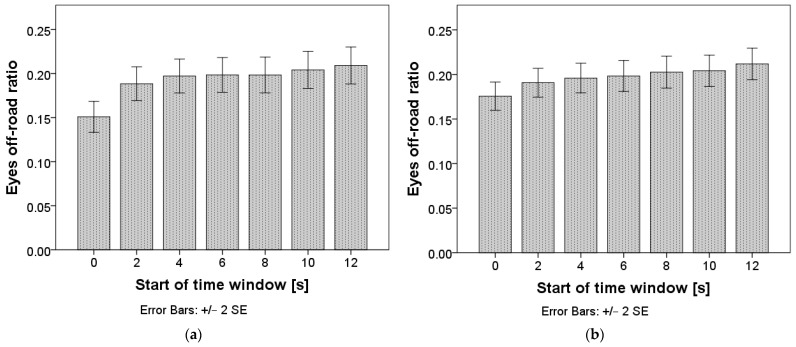
Eyes off-road ratio after the takeover. Chart (**a**) represents calculations with a window length of two seconds; chart (**b**) represents calculations with a window length of five seconds.

Repeated measures ANOVA confirmed that differences in eyes off-road ratio exist between time windows: F(5.4, 3506.1) = 6.12, *p* < 0.001 for time windows of two seconds, and F(2.8, 1797.8) = 4.27, *p* = 0.006 for time windows of five seconds. The mean values are presented in Figure 6, and the pairwise comparisons can be found in Appendix A, Table A1 and Table A2. E-OFF stabilized in the time interval starting two seconds after the TO for both window lengths.

**Figure 7:**
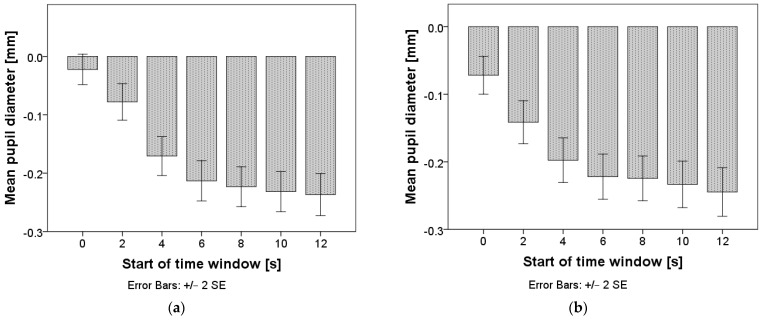
Mean pupil diameter after the takeover. Chart (**a**) represents calculations with a window length of two seconds; chart (**b**) represents calculations with a window length of five seconds.

Repeated measures ANOVA confirmed that differences in pupil diameter exist between time windows: F(3.5, 2150.0) = 143.9, *p* < 0.001 for time windows of two seconds, and F(2.1, 1311.5) = 129.8, *p* < 0.001 for time windows of five seconds. The mean values are presented in Figure 7, and the pairwise comparisons can be found in Appendix A, Table A1 and Table A2. Pupil diameter stabilized in the time interval starting six seconds after the TO for both window lengths. When using a window length of five seconds, the pupil diameter started dropping again in the interval starting twelve seconds after the TO.

**Figure 8:**
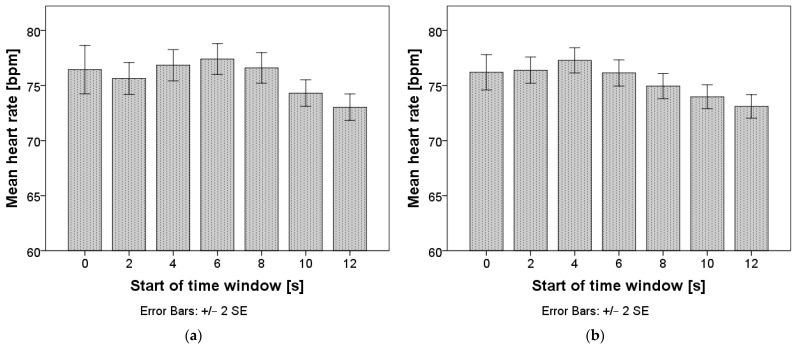
Mean heart rate after the takeover. Chart (**a**) represents calculations with a window length of two seconds; chart (**b**) represents calculations with a window length of five seconds.

Repeated measures ANOVA confirmed that differences in heart rate exist between time windows: F(3.6, 150.1) = 2.76, *p* = 0.035 for time windows of two seconds, and F(2.6, 263.7) = 15.8, *p* < 0.001 for time windows of five seconds. The mean values are presented in Figure 8, and the pairwise comparisons can be found in Appendix A, Table A1 and Table A2. When using a window length of two seconds, no statistically significant differences between data in adjacent time intervals were observed. When using a window length of five seconds, the HR only started dropping in the interval starting eight seconds after the TO and stabilized again in the interval starting ten seconds after the TO.

**Figure 9:**
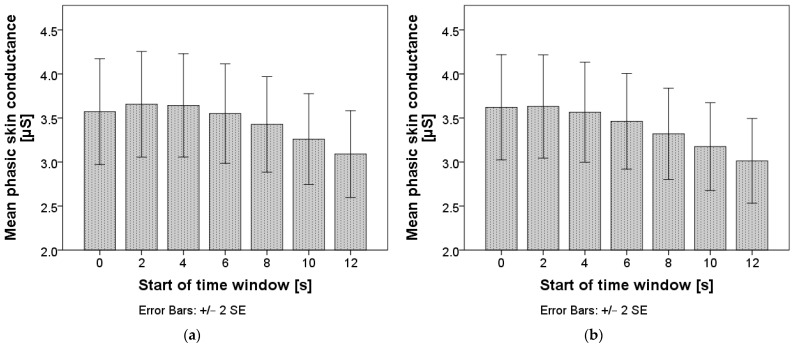
Mean phasic skin conductance after the takeover. Chart (**a**) represents calculations with a window length of two seconds; chart (**b**) represents calculations with a window length of five seconds.

Repeated measures ANOVA confirmed that differences in phasic skin conductance exist between time windows: F(1.2, 708.6) = 9.90, *p* = 0.001 for time windows of two seconds, and F(1.1, 653.7) = 12.6, *p* < 0.001 for time windows of five seconds. The mean values are presented in Figure 9, and the pairwise comparisons can be found in Appendix A, Table A1 and Table A2. Phasic skin conductance only started dropping in the time interval starting six seconds after the TO and did not stabilize while measuring for both window lengths.

## 4. Discussion

This study aimed to investigate two primary research questions: (1) How long after takeover could stabilization be achieved, and could it occur before reaching the system limit (e.g., impact); and (2) Do physiological signals exhibit a similar stabilization time to driving-related parameters, and does a driver remain stressed or aroused longer after takeover than predicted from vehicle parameters? Our findings provide insights into these research questions, shedding light on the temporal and cognitive dynamics underlying the stabilization process in takeover scenarios in conditionally automated driving environments.

Regarding the first research question, our results indicated that the stabilization time following takeover (TO) varied for different parameters. Notably, deceleration and eyes off-road ratio stabilized approximately two seconds after TO, then pupil diameter stabilized approximately six seconds after TO, winding stabilized approximately eight seconds after TO, speed stabilized eight to ten seconds after TO, heart rate stabilized more than ten seconds after TO, and phasic skin conductance did not stabilize at all in both the two-second and five-second intervals.

Deceleration being one of the first parameters to stabilize indicates that the drivers probably applied the brake pedal to take over the vehicle, slow down a bit, and then stopped braking, which is in line with the findings of Gruden et al. [47]. The E-OFF ratio also stabilized approximately two seconds after TO, indicating a rapid adjustment of visual attention after the transition, in line with Stephenson et al. [69]. Pupil diameter, often considered a direct indicator of driver cognitive load [66], showed stabilization starting several seconds after TO. It should be noted that pupil diameter stabilizing at a lower value than at TO could reflect lower cognitive load after TO or adaptation to changes in light conditions [75,104]. As the driver was performing a secondary task before TO, the driver’s gaze was directed off the driving simulator screen, which could have different illumination than when looking at the screen (road). However, as pupil constriction due to sudden illumination changes occurs in fractions of a second [105], we believe that our measurements reliably reflect the decrease in mental load that stabilized six seconds after TO. After the decrease in mental load, the stabilization times of winding and speed show that driving-related parameters stabilize about eight to ten seconds after TO. This is less than the stabilization reported by Kim et al. [55], but is consistent with Riahi Samani and Mishra [57], who found that most information can be obtained by observing the first ten seconds following TO. It should be noted that they measured stabilization based on a different method. As Kim et al. instructed drivers to say “stable” when they felt stable, it is reasonable that this self-reported stabilization is longer, as the vehicle should already be stable for some time before the driver considers it stable. However, this is still more than the usually adopted TORlt of six seconds [24], implying that drivers might not be able to stabilize the vehicle in time, e.g., before impact with an obstacle. Therefore, using longer TORlt, as proposed by Tan and Zhang [23], might be beneficial. As the driver should have enough time to react and stabilize to perform a qualitative TO, the sum of reaction and stabilization times should be lower than or at least similar to the TORlt. Heart rate and phasic skin conductance did not begin to decrease until approximately six to eight seconds after TO, indicating a prolonged arousal state following TO and vehicle-related stabilization.

Addressing the second research question, our findings suggest that physiological signals do not necessarily exhibit a similar stabilization time as driving-related parameters. While driving-related parameters such as winding and speed stabilized within a few seconds after TO, physiological signals, especially HR and SC, demonstrated more prolonged stabilization periods, as proposed by Gruden et al. [76]. HR and SC exhibited variations between adjacent time intervals, indicating a longer period of arousal following TO than predicted from vehicle parameters alone. HR stabilized only in the last observed time window when using the window length of five seconds, in line with Kerautret et al. [75], while SC did not stabilize during the observed duration. Therefore, we propose longer observations of physiological parameters in future studies to reliably detect drivers’ arousal stabilization. We suspect that the drivers remain aroused while being able to reasonably drive the vehicle, i.e., after achieving vehicle-related stabilization. However, if another unforeseen situation occurs while being stressed, drivers might react worse due to still being under the influence of the last TO. This discrepancy highlights the importance of considering both physiological and driving-related parameters when assessing drivers’ arousal and stress levels during takeover scenarios.

Additionally, vehicle speed exhibited a different profile than other parameters, which monotonously stabilized after TO. It initially dropped and showed an earlier stabilization with a five-second interval occurring two seconds after TO, followed by an increasing trend and a subsequent stabilization eight seconds after TO. The initial drop could be explained by the immediate deceleration at TO, which settled afterwards. Speed, deceleration, and heart rate also exhibited variations between the lengths of the time windows. It should be noted that the deceleration at the interval length of two seconds was probably not found significant, as there were not many TO attempts (only about 10%) where drivers gradually decelerated and therefore deceleration data could be calculated. The longer size, on the other hand, resulted in overlapping time windows that contained more data samples. Something similar could be stated for HR data measured with the E4 wristband. As the driver is moving during a TO, many HR samples were not available due to motion artifacts [71]. Since heartbeats are produced about once per second, there is a high probability that data were not available in most of the two-second intervals. According to our results, this could, however, not be stated for the five-second intervals.

Therefore, it is essential to acknowledge some limitations of the study. (1) As previously mentioned, the different illumination of the environment and the driving simulator screen may have affected pupil diameter measurements [104]. (2) There were many missing samples for deceleration and heart rate. Future studies could interpolate the missing values or use some other tools for analysis. (3) The experiments were conducted in a controlled high-fidelity driving simulator environment. While driving simulators offer several advantages, including controlled experimental conditions and enhanced safety, they may not fully replicate the stress, distraction, and other real-world driving conditions. However, user studies with potentially dangerous scenarios could never be conducted on the road without exposing participants to actual risk. Moreover, simulators allow us to systematically manipulate variables, assess driver responses, and collect detailed data that are difficult to obtain in on-road studies. Therefore, despite their limitations, driving simulators are a valuable tool for studying takeover performance. (4) Additionally, the study focused on a limited set of physiological parameters, and further investigation of additional measures of cognitive workload and emotional state could provide a more comprehensive understanding of stabilization time.

Next, we open up some interesting discussion topics related to stabilization time. These considerations could be further investigated by readers, as a deeper analysis would exceed the sole purpose of this manuscript. Our analysis could be further generalized by drawing on and comparing the results of other researchers’ TO studies or tailored to individual technological systems. While our study focused primarily on identifying factors that reveal stabilization time, future research could explore interventions or strategies aimed at shortening the time it takes for drivers to regain full control after a takeover, similar to studies conducted to find the optimal TO user interface [35,37,46]. For example, Petermeijer et al. showed that auditory TOR stimuli provoked the fastest response [30]. The guidelines by Naujoks et al. [106] also provide a way to design an optimal user interface and show which features should be included. Possible approaches include the development of advanced automation interfaces (integration of real-time feedback mechanisms, adaptive displays, and ergonomic design to support rapid and effective TO), driver training programs, or predictive algorithms to improve driver readiness and responsiveness to takeover events. The datasets we used consisted of takeover attempts with the same lead time of five seconds, as this is considered optimal for takeover requests in the literature [21]. However, future research could dive further into the relationship between takeover lead time and stabilization time and how different lead times affect driver adaptation and performance during takeover events. The effects of the urgency of the takeover scenario and the driver’s secondary task on overall TO performance have been studied in depth, but their effects on stabilization time have not yet been adequately addressed. The aim of this paper is to determine the stabilization time in a general takeover scenario. In the dataset used, participants were allowed to perform any type of secondary task at their own discretion. Although the investigation of the influence of scenario complexity is beyond the scope of the present manuscript, it could be useful for the development of adaptive, context-aware, and robust automated driving systems.

## 5. Conclusions

Our study contributes to the understanding of stabilization time in takeover scenarios in conditionally automated driving environments. We have shown that driving-related stabilization can be achieved approximately eight to ten seconds after the TO, which is more than the commonly assumed TOR lead time. We also demonstrated that physiological signals, particularly heart rate and phasic skin conductance, exhibited prolonged stabilization periods, indicating that drivers remain aroused even after driving-related stabilization is achieved.

Future studies should extend the observation period after TO to reliably determine the stabilization time of phasic skin conductance, which did not stabilize during our observations. In addition, a more thorough analysis of the missing values for heart rate and deceleration should be performed. Ultimately, a model of drivers’ mental states throughout the TO process could be derived from this and similar studies to better understand the process. The diversity of drivers could be taken into account by clustering drivers based on demographic data and analyzing different driver profiles. To improve the generalizability of the results, future analysis could combine data from many TO studies conducted by research groups around the world to obtain valid comparisons under many different conditions and using different technologies. Using artificial intelligence and machine learning techniques, predictive algorithms could anticipate upcoming takeover events and proactively assist drivers to reduce stabilization time. By addressing these future research directions, we can further improve our understanding of stabilization time and accelerate progress towards a future where automated vehicles seamlessly coexist with human drivers, ushering in a new era of mobility characterized by enhanced safety, efficiency, and accessibility.

## Data Availability

The data are available upon request to the corresponding author.

## Appendix A

Pairwise comparisons for the dependent variables in the analysis.

**Table A1:** Pairwise comparisons for window length of two seconds.

Start of Time w. [s]		Winding [rad]		Speed [m/s]		Eyes Off-Road Ratio		Pupil Diameter [mm]		Heart Rate [bpm]		Phasic Skin Conductance [µS]	
(I)	(II)	Mean Diff. (II–I)	*p*-Value	Mean Diff. (II–I)	*p*-Value	Mean Diff. (II–I)	*p*-Value	Mean Diff. (II–I)	*p*-Value	Mean Diff. (II–I)	*p*-Value	Mean Diff. (II–I)	*p*-Value
0	2	0.093 *	<0.001	−1.579 *	<0.001	0.038 *	0.001	−0.062 *	<0.001	0.21	1.000	0.080	0.071
	4	0.148 *	<0.001	−1.875 *	<0.001	0.049 *	<0.001	−0.155 *	<0.001	−0.16	1.000	0.067	1.000
	6	0.178 *	<0.001	−1.585 *	<0.001	0.047 *	<0.001	−0.197 *	<0.001	0.96	1.000	−0.019	1.000
	8	0.195 *	<0.001	−1.273 *	<0.001	0.044 *	0.002	−0.204 *	<0.001	−0.40	1.000	−0.124	1.000
	10	0.209 *	<0.001	−1.035 *	<0.001	0.051 *	<0.001	−0.216 *	<0.001	−1.26	1.000	−0.267	0.492
	12	0.213 *	<0.001	−0.962 *	<0.001	0.055 *	<0.001	−0.221 *	<0.001	−2.11	1.000	−0.391	0.153
2	4	0.055 *	<0.001	−0.296 *	0.002	0.010	1.000	−0.093 *	<0.001	−0.37	1.000	−0.013	1.000
	6	0.084 *	<0.001	−0.006	1.000	0.008	1.000	−0.134 *	<0.001	0.75	1.000	−0.099	0.963
	8	0.102 *	<0.001	0.306	1.000	0.006	1.000	−0.142 *	<0.001	−0.61	1.000	−0.204	0.165
	10	0.115 *	<0.001	0.544	0.090	0.013	1.000	−0.153 *	<0.001	−1.47	1.000	−0.347 *	0.021
	12	0.119 *	<0.001	0.617	0.114	0.017	1.000	−0.159 *	<0.001	−2.32	0.788	−0.471 *	0.010
4	6	0.030 *	<0.001	0.290 *	0.001	−0.002	1.000	−0.041 *	<0.001	1.12	1.000	−0.086 *	0.031
	8	0.047 *	<0.001	0.602 *	<0.001	−0.004	1.000	−0.049 *	<0.001	−0.23	1.000	−0.191 *	0.015
	10	0.061 *	<0.001	0.840 *	<0.001	0.003	1.000	−0.061 *	<0.001	−1.09	1.000	−0.333 *	0.002
	12	0.065 *	<0.001	0.913 *	0.001	0.006	1.000	−0.066 *	<0.001	−1.95	1.000	−0.457 *	0.002
6	8	0.018 *	0.008	0.311 *	0.001	−0.002	1.000	−0.008	1.000	−1.36	0.713	−0.105 *	0.017
	10	0.031 *	<0.001	0.550 *	0.002	0.005	1.000	−0.019	0.926	−2.21	0.123	−0.248 *	0.002
	12	0.035 *	0.001	0.623 *	0.025	0.008	1.000	−0.024	0.601	−3.07 *	0.026	−0.372 *	0.002
8	10	0.014	0.153	0.238 *	0.049	0.007	1.000	−0.012	1.000	−0.86	1.000	−0.143 *	0.001
	12	0.018	0.349	0.311	0.664	0.011	1.000	−0.017	1.000	−1.72	1.000	−0.267 *	0.001
10	12	0.004	1.000	0.073	1.000	0.004	1.000	−0.005	1.000	−0.86	1.000	−0.124 *	0.007

* The mean difference is significant at the 0.05 level.

**Table A2:** Pairwise comparisons for window length of five seconds.

Start of Time w. [s]		Winding [rad]		Speed [m/s]		Deceleration [m/s^2^]		Eyes Off-Road Ratio		Pupil Diameter [mm]		Heart Rate [bpm]		Phasic Skin Conductance [µS]	
(I)	(II)	Mean Diff. (II–I)	*p*-Value	Mean Diff. (II–I)	*p*-Value	Mean Diff. (II–I)	*p*-Value	Mean Diff. (II–I)	*p*-Value	Mean Diff. (II–I)	*p*-Value	Mean Diff. (II–I)	*p*-Value	Mean Diff. (II–I)	*p*-Value
0	2	0.067 *	<0.001	−0.763 *	<0.001	−0.155 *	<0.001	0.016 *	0.006	−0.072 *	<0.001	−0.29	1.000	0.012	1.000
	4	0.105 *	<0.001	−0.709 *	<0.001	−0.238 *	<0.001	0.021 *	0.049	−0.127 *	<0.001	−0.60	1.000	−0.048	1.000
	6	0.128 *	<0.001	−0.417 *	0.014	−0.202 *	0.001	0.020	0.331	−0.150 *	<0.001	−1.25	1.000	−0.150	0.757
	8	0.141 *	<0.001	−0.186	1.000	−0.126	0.714	0.023	0.152	−0.158 *	<0.001	−2.40 *	0.014	−0.277	0.109
	10	0.149 *	<0.001	−0.016	1.000	−0.115	1.000	0.025	0.072	−0.166 *	<0.001	−3.65 *	<0.001	−0.400 *	0.035
	12	0.152 *	<0.001	0.173	1.000	−0.143	0.317	0.032 *	0.007	−0.179 *	<0.001	−3.80 *	<0.001	−0.515 *	0.016
2	4	0.038 *	<0.001	0.054	1.000	−0.083	0.422	0.005	1.000	−0.056 *	<0.001	−0.31	1.000	−0.059	0.380
	6	0.061 *	<0.001	0.346 *	0.029	−0.047	1.000	0.004	1.000	−0.079 *	<0.001	−0.96	0.751	−0.162 *	0.044
	8	0.074 *	<0.001	0.577 *	0.005	0.023	1.000	0.007	1.000	−0.086 *	<0.001	−2.11 *	0.001	−0.289 *	0.009
	10	0.081 *	<0.001	0.748 *	0.003	0.040	1.000	0.010	1.000	−0.094 *	<0.001	−3.36 *	<0.001	−0.412 *	0.005
	12	0.084 *	<0.001	0.936 *	0.001	0.012	1.000	0.017	1.000	−0.108 *	<0.001	−3.51 *	<0.001	−0.527 *	0.003
4	6	0.023 *	<0.001	0.292 *	<0.001	0.036	1.000	−0.001	1.000	−0.023 *	<0.001	−0.65	0.663	−0.102 *	0.007
	8	0.036 *	<0.001	0.523 *	<0.001	0.112	0.258	0.002	1.000	−0.031 *	<0.001	−1.80 *	<0.001	−0.230 *	0.002
	10	0.044 *	<0.001	0.693 *	0.001	0.123	0.245	0.004	1.000	−0.039 *	0.001	−3.05 *	<0.001	−0.352 *	0.002
	12	0.046 *	<0.001	0.882 *	0.001	0.095	0.927	0.011	1.000	−0.052 *	<0.001	−3.20 *	<0.001	−0.468 *	0.001
6	8	0.013 *	0.001	0.231 *	0.012	0.076	0.847	0.003	1.000	−0.008	1.000	−1.15 *	<0.001	−0.127 *	0.001
	10	0.020 *	0.004	0.402 *	0.031	0.087	0.832	0.006	1.000	−0.016	1.000	−2.39 *	<0.001	−0.250 *	0.001
	12	0.023 *	0.019	0.590 *	0.013	0.059	1.000	0.012	1.000	−0.029 *	0.018	−2.55 *	0.002	−0.365 *	0.001
8	10	0.008	0.303	0.170	0.223	0.011	1.000	0.002	1.000	−0.008	1.000	−1.25 *	0.007	−0.123 *	0.002
	12	0.011	1.000	0.359	0.062	−0.17	1.000	0.009	1.000	−0.021 *	0.021	−1.40	0.191	−0.238 *	0.002
10	12	0.003	1.000	0.188 *	0.048	−0.028	1.000	0.007	1.000	−0.013 *	0.003	−0.16	1.000	−0.115 *	0.002

* The mean difference is significant at the 0.05 level.
